# Obituary Notice. Prof. Thomas Walley

**Published:** 1894-12

**Authors:** 


### OBITUARY NOTICE.

Professor Thomas Walley, Principal of the Royal (Dicks) Veterinary College, Edinburgh, Scotland, died on the 10th instant (December) at La Haule, Jersey (England). His remains were conveyed to London, and the funeral took place at Highgate Cemetery, London, N., on the 15th instant. A number of friends, private and professional, attended, deeply grieved at the loss of such a dear friend.

Thomas Walley graduated from the Royal Veterinary College of London, at the Royal College of Veterinary Sergeons, Red Lion Square, on the 29th day of April, 1863. He embarked in practice in the North of England, and from his painstaking in the general routine of practice together with his assiduity as a student, he was selected to the vacant chair at Dick's College on the resignation or retirement of Professor Williams. As the principal of that College, he has done faithful work for the institution and its students, being held in the highest esteem by all with whom he came in contact, through his genial and candid bearing.

The four "Bovine Scourges" from his pen have been read by most every veterinary surgeon and inspector. The painstaking exhibited in these volumes is characteristic of the man in all his labors. He was a stanch friend to all domesticated animals, ever ready to defend them from acts of cruelty.

For some weeks Professor Walley's health had been failing, hence his visit to Jersey for an entire change of climate.

The profession has sustained a great loss, and so has the College over which he presided for so many years. We think it was in 1875 that Prof. Walley was elected to the vacant chair; thus twenty years of active service were passed by him prior to his death. * * * It is said: "There is no such thing as death—

In nature nothing dies;
From each sad remnant of decay
Some forms of life arise.

The little leaf that falls,
All brown and sere to earth,
Ere long will mingle with the buds
That give the flower its birth."

